# Whole genome sequencing of Gyeongbuk Araucana, a newly developed blue-egg laying chicken breed, reveals its origin and genetic characteristics

**DOI:** 10.1038/srep26484

**Published:** 2016-05-24

**Authors:** Hyeonsoo Jeong, Kwondo Kim, Kelsey Caetano-Anollés, Heebal Kim, Byung-ki Kim, Jun-Koo Yi, Jae-Jung Ha, Seoae Cho, Dong Yep Oh

**Affiliations:** 1 Department of Animal Sciences, University of Illinois, Urbana, IL 61801, USA; 2 Interdisciplinary Program in Bioinformatics, Seoul National University, Kwan-ak St. 599, Kwan-ak Gu, Seoul, South Korea 151-741, Republic of Korea; 3 C&K genomics, Main Bldg. #514, SNU Research Park, Seoul 151-919, Republic of Korea; 4 Department of Agricultural Biotechnology, Animal Biotechnology Major, and Research Institute for Agriculture and Life Sciences, Seoul National University, Seoul 151-921, Korea; 5 Department of Agricultural Biotechnology, Seoul National University, Kwan-ak St. 599, Kwan-ak Gu, Seoul 151-742, Republic of Korea; 6 Gyeongsangbuk-do Livestock Research Institute, 186, Daeryongsan-ro, Anjung-myon, Yeoungju, Gyeongsangbuk-do, Republic of Korea

## Abstract

Chicken, *Gallus gallus*, is a valuable species both as a food source and as a model organism for scientific research. Here, we sequenced the genome of Gyeongbuk Araucana, a rare chicken breed with unique phenotypic characteristics including flight ability, large body size, and laying blue-shelled eggs, to identify its genomic features. We generated genomes of Gyeongbuk Araucana, Leghorn, and Korean Native Chicken at a total of 33.5, 35.82, and 33.23 coverage depth, respectively. Along with the genomes of 12 Chinese breeds, we identified genomic variants of 16.3 million SNVs and 2.3 million InDels in mapped regions. Additionally, through assembly of unmapped reads and selective sweep, we identified candidate genes that fall into heart, vasculature and muscle development and body growth categories, which provided insight into Gyeongbuk Araucana’s phenotypic traits. Finally, genetic variation based on the transposable element insertion pattern was investigated to elucidate the features of transposable elements related to blue egg shell formation. This study presents results of the first genomic study on the Gyeongbuk Araucana breed; it has potential to serve as an invaluable resource for future research on the genomic characteristics of this chicken breed as well as others.

Chicken, *Gallus gallus*, is valuable not only as a food source but also as a model organism for scientific research^1^. In the past thousands of years, hundreds of chicken breeds have diverged under natural and artificial selection in a wide variety of circumstances. As a result, chickens have undergone significant phenotypic differentiation in body size, plumage, egg color, and flying ability^2^.

The Gyeongbuk Araucana (GA) domestic chicken is a hybrid breed developed in Gyeongbuk, Korea by crossing the Golden Duckwing Araucana and the White Leghorn, two breeds with very distinct characteristics. The White Leghorn, a small breed with a rump, is renowned for its prolific egg-laying as well as a good feed-to-egg conversion ratio. Meanwhile, the Golden Duckwing Araucana is a similarly sized small rumpless and tufted breed that produces blue-shelled eggs at a relatively slow rate. These two breeds were crossed to produce a Korean chicken variety which possesses qualities favorable to commercial poultry production from both parent breeds, including the blue shell tint of the eggs they lay and high egg production rate. Although both the Golden Duckwing Araucana and White Leghorn are small breeds, GA resulting from the cross are extremely large and can fly well. Additionally, GA chickens display a combination of phenotypic traits from both parents: a rump but no ear tufts (Fig. 1). Although GA is a relatively new breed, it has already been registered in the Domestic Animal Diversity Information System (DAD-IS) of the FAO (Food and Agriculture Organization).

Analysis of genetic information and patterns can be useful for discovering the origin of specific breed or detecting specific traits within breeds. Recent phylogenetic analyses using various genetic information revealed the origin of the domesticated chicken^3^ and the Korean native chicken (KNC)^4^. Additionally, genetic variants present between several chicken breeds have been utilized to support the characterization of specific traits in chicken breeds^5^,^6^,^7^. In this way, a wide range of genomic studies on domestic animals, and in chickens specifically, have been conducted to investigate the genetic architecture of these species. However, no such study has been performed in GA. For this reason, we performed whole genome sequencing on GA chickens and additionally performed whole genome sequencing of Leghorn (LH) and KNC. The whole genome paired-end reads for other 12 chicken breeds were also obtained from the sequence read archive (SRA) in EMBL-EBI database. Using genomic information from 28 chickens, we identified candidate genomic characteristics which may be related to Gyeongbuk Araucana’s phenotypic traits. Our results also confirmed the results of previous studies related to blue egg formation^8^,^9^. This study is the first of its kind to report a comprehensive view of the GA chicken breed at a genomic level.

## Results and Discussion

### Short read alignment and variant calling

The whole genomes of 9 chicken samples (3 GA, 3 LH, and 3 KNC) were sequenced to an average depth of 11.4 fold , with 9,422,388,891 bp in a total of 1,095,144,380 reads. Short sequencing reads of each breed were aligned to the chicken reference genome (*Galgal 4.75*) from the Ensembl database with an overall alignment rate of 97.81%. The average depth for the overall dataset of 28 Chickens was 17.2 fold, and the mapping rate in different breeds varied from 95.89% to 98.42%. (Table S1). We then identified 16,342,621 single nucleotide variants (SNVs) and 2,254,900 Indels in all 28 samples after removing PCR duplicates and recalibrating base quality of sequencing reads. To obtain variants for population analyses, we removed the variants supposing missing data present in any of the 28 individuals or in non-chromosomal regions. Although 7,122,335 SNVs (43.58%) had been identified previously (ftp://ftp.ensembl.org/pub/release-83/variation/vcf/gallus_gallus/Gallus_gallus.vcf.gz), we defined 9,220,286 SNVs as candidates for novel variants (56.42%). The detailed results of variant calling and functional annotation are shown in Figures S1 and S2, Tables 1 and S2.

### Population differentiation of 28 chickens

**Figure 2:**
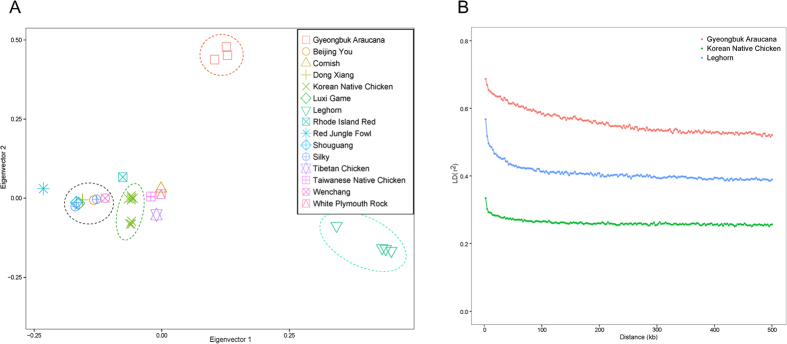
Population stratification of various chicken breeds. (**A**) Results of principal component analysis (PCA) of 15 chicken breeds. PC1 (x-axis) versus PC2 (y-axis). PC1 and PC2 represent 12.85% and 11.08% of the total variance in the PCA, respectively. (**B**) Linkage disequilibrium (LD) pattern of GA, KNC, and LH.

To understand the population differentiation, structure, and relationship between various breeds more deeply, we performed several analyses based on the high quality variants. First, principle component analysis (PCA) was performed to distinguish 15 different breeds using two main components explaining the dispersion of samples (Fig. 2A). GA, LH, and KNC were well divided by breed using Eigenvector 1 (12.85% of the total variance) and Eigenvector 2 (11.08% of the total variance) for PCA. Results revealed clear structural differences between populations. We further measured kinship coefficient, also known as coefficient of coancestry, and IBS (identical by state) to measure the pairwise relationship between each sample (Figure S3). The relationships were clear in GA chickens but not in one of the LH samples and not in most of the KNC samples. From the results of three analyses, it has been clearly demonstrated that GA genetic profile is overall significantly distinct from that of other chicken breeds.

### Gene prediction using unmapped reads

To identify breed-specific genes in GA, we assembled unmapped reads into contigs. However, they were inadequate for detection of gene regions given their short length. Thus, we performed whole genome assembly using all short read sequences (Table S3) and then matched the unmapped assembled contigs to the assembled whole genome contigs using BLAST. 424, 459, and 375 putative peptides from the matched contigs of 3 GA samples (GA1, GA2, and GA3, respectively) were annotated. Of the total putative genes, 61 were shared between all GA samples. These genes were predominantly related to growth (such as ‘epithelium development (4 genes, p-value = 0.01)’, ‘cell cycle (6 genes, p-value = 0.03)’, and ‘structural molecule activity (5 genes, p-value = 0.03)’). Additionally, KEGG (Kyoto Encyclopedia of Genes and Genomes) pathway analysis was performed. Results revealed that several genes were involved in the Notch signaling pathway that is responsible for functions relating to cardiac valve homeostasis^10^, stabilization of angiogenesis^11^, and neuronal function and development^12^. Given GA’s large body size, it makes sense that genes related to growth were predominantly identified. In addition, as the process of gene prediction was based on sequence similarity between assembled contigs and reference genes, the predicted genes are not regarded as totally novel genes. On the other hand, alignments of unmapped reads to the regions with genes implicates that the genes in these particular breeds have considerable variants, so sequenced reads couldn’t be mapped to the reference genome.

### Selective sweep signal in GA

Compared to other breeds, GA display a consistently high level of linkage disequilibrium over varying distances of the genome (Fig. 2B). This may reflect the fact that only a small number of the Golden Duckwing Araucana, one of the GA’s parent breeds, was imported to Korea causing GA chickens to have a low level of genetic diversity.

**Figure 3:**
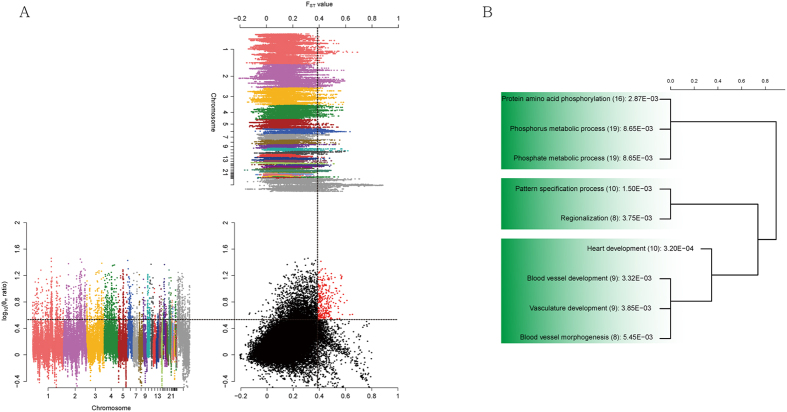
Manhattan plot of distribution of pairwise nucleotide variation (log_2_ (*θ*_*π*_ ratio)) and genetic differentiation (Weir and Cockerham’s F_ST_).

To find distinctly selected genomic regions in GA, we measured genome-wide variation between GA and KNC (Fig. 3A). KNC is a well-suited breed for comparison since it lives geographically close to and is genetically different from GA. In order to overcome the limitation of small sample size, we detected candidate genomic regions using the overlap of top 5% regions of pairwise nucleotide variation (log_10_ (*θ*_*π*_ ratio)) and genetic differentiation (Weir and Cockerham’s F_ST_) as significant regions following protocol used in previous studies^13^. The F_ST_ statistic is less sensitive to small sample size if sufficient variant loci are examined^14^,^15^. From the chicken genome, 46,354 windows were used to detect selective sweep since these windows contain more than 50 SNPs (92.9% of the genome). We identified 223 genes as a positive signature of the each statistic (log_10_ (*θ*_*π*_ ratio) >3.378, F_ST_ > 0.387). Although the Z chromosome is a highly conserved region (Figure S2), most of the selected regions were located on the Z chromosome (17.4%). Meanwhile, the selected genes were predominantly related to cardiac development and morphogenesis (Fig. 3B). In avian species, flying has been found to associate with the cardiovascular system; due to the physiological strain resulting from flying, most flying birds have strong cardiac muscles^16^. *MYLK2*, identified as significant from our analyses, has previously been established as important in cardiac muscle tissue morphogenesis as well as fast-twitch skeletal muscle fiber contraction, both of which are required for quick bursts of activity such as flight^17^. Similarly, we identified several genes (*BCL2L1*, *GSS*, *SCARA3*, *ERBB4*) associated with response to oxidative stress^18^,^19^. As chickens only fly at high intensity in short bursts, it is particularly important for them to have a mechanism to protect their body from the damage of free radicals.

Using the same procedure, we compared LH and KNC, using KNC as a reference, in order to identify positively selected regions in LH. From the results, we identified positive selection regions which overlap between LH and GA; identification of genomic regions responsible for egg production in the LH breed may help decipher the genetic basis of GA’s superior egg laying performance. We found that all overlapped windows were located on the Z chromosome. Among the genomic area, a window at 32.1 ~ 32.2 Mbp was found to be significantly related with egg production in the chicken QTL region^20^. It was also involved within the previously reported QTL region related to maternal age at the first egg production^21^. Results of our analyses provide important insight into the genomic features of a breed which has not been investigated previously. However, as a relatively small sample size was used in the present study, future genomic studies on this breed using a larger sample size may help further elucidate the underlying genetic factors behind this novel and unique breed.

### Identification of transposable element variants (TEV)

**Figure 4:**
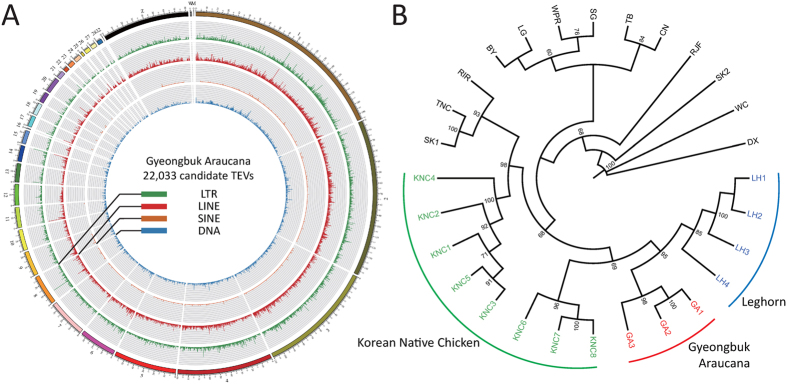
Results of analyses using transposable element variants (TEVs). (**A**) A circular plot for distribution of TEVs on the genome of GA. Four types of TE are represented by different colors (LTR : green, LINE : red, SINE: orange, and DNA: blue). (**B**) A clustering tree based on LTR presence patterns for 28 chicken genomes. Three groups for LH, GA, and KNC are well-defined. The neighbor-joining tree using whole genome variants and other TE types (LINE, SINE, and DNA) are shown in Figure S4.

Using Retroseq software^22^, we obtained a total of 412,208 candidate TEVs from 9 genomic samples (GA, LH, KNC) and 19 public genome sequences. Most of the TEVs (~250,000) were located in the intergenic region, while only 149 TEVs were found in the exon region (Table S4). From GA, we identified 22,033 candidate TEVs which differed from the chicken reference genome (*Galgal 4.75*). Most of the TEVs were annotated as intergenic sequences and only 11 TEVs as exon sequence (Table 2 and Fig. 4A). As found in previous studies^23^, the majority of TEVs belonged to the CR1 families of LINEs (CR1 : 10,524, LINE : 10,676, LTR : 8,587, DNA : 2,611, SINE : 159), which is consistent in other non-GA chicken breeds as well.

### Clustering analysis based on TE variants identified from 28 chicken genomic samples

Using TEV information from 9 chicken genome along with 19 public genome sequences, we performed clustering analysis based on the pattern of TE presence in 28 chicken genomes. Several other studies have constructed phylogenetic trees based on the TE presence pattern in primates^24^,^25^,^26^,^27^. These studies on the relationship between species showed that incongruent TE insertion sites can be caused by several factors, including incomplete lineage sorting and hybridization between species. Considering these factors within species and mating-free environment, we estimated that these factors would considerably impact TE presence patterns and consequently cause a confounding result. As predicted, we obtained results similar to those estimated for TE types like LINE, SINE, and DNA transposons (Figure S4). However, for LTR, the constructed tree was similar to that of SNP based-clustering, which roughly segregates whole individuals by breed (Figs 4B and S4A). This result indicates that LTR polymorphism might be used as a marker for revealing the relationship of relatively close organisms and is consistent with those from a previous study on the effects of retroviral insertions on phenotypic traits of breeds^28^ as well as blue egg shell formation^10^,^27^.

The clustering results based on the LTR presence pattern are shown in Fig. 4B. Like the SNP-based tree, GA and LH were clustered into one group and close to KNC, which was expected given the the origin of GA as a result of hybridization between the Golden Duckwing Araucana and White leghorn.

### Candidate retroviral insertions specific to GA chickens

The genetic determination of blue egg shell coloration has been identified in Araucana chickens; it has been revealed that EAV-HP insertion promotes the expression of *SLCO1B3* gene in the uterus of the oviduct in Araucana chickens, which causes blue egg shell formation^9^,^29^. Our results identified retroviral insertions from three GA genomes in *SLCO1B3* gene equally. Dong Xiang (DX) chickens are another blue-egg laying breed^29^; EAV-HP insertion was also identified in the DX genome sequence adjacent to the insertion of the *SLCO1B3* gene in GA, which indicates that GA and DX share genetic characteristics with other breeds that lay blue eggs. All of these results are consistent with previous reports^9^,^29^.

As we detected the presence of TE insertions using multiple TE probe sequences at the breakpoint, it was necessary to verify that the sequences of TE insertions are identical. In order to validate the presence of EAV-HP sequence, we carried out local de novo assembly with Velvet^30^ using the reads mapped within 1000 upstream and downstream of the candidate breakpoint. From this process, we obtained several contigs and aligned these with the TE probes we used for TE identification. Next, we conducted multiple sequence alignment over sequences recovered from the above process of several individuals, and retrieved partial conserved TE sequences (142 bp) from 3 GA and DX genomes. Collectively, the commonly identified insertion of EAV-HP in three GA chickens was identical to that of previous experimental studies^9^,^29^.

Retroviral insertion can influence the transcription of genes in many ways. Several studies on mice found that the effects of intronic retrovirus insertion on the transcription of the resident gene result in an alteration of the ratios of the splice variants by premature transcription, either by providing a cryptic promoter or by altering splicing. In the case of blue egg shell formation, upstream insertion of resident genes can influence expression of the gene^31^. Consequently, these transcriptional effects can modify phenotypic traits, which has been shown in chickens^28^ as well as mice^32^,^33^.

Here, we propose candidate TEVs specific to GA chickens. We identified one LTR insertion specific to GA within *SUCLG1* gene. This insertion was located in the 3′ UTR region of the gene and we retrieved the partial sequence of 188 bp conserved in all three GA genomes. *SUCLG1* gene has been investigated for its role as an α subunit of succinate-CoA ligase-encoding gene, which forms a complex with nucleoside diphosphate kinase and plays an important role in the salvage of deoxyribonucleotides for mitochondrial DNA synthesis^34^. *SCN5A* also contained a LTR insertion identified only in GA within the intron region (136 bp conserved sequence). *SCN5A* is a gene encoding cardiac-specific voltage-gated sodium channel and known to be related to many cardiovascular diseases^35^,^36^. This result was consistent with identification of heart and muscle development-related genes within selection signal.

## Conclusions

In this study, we conducted whole genome sequencing and analyzed genomic variants in order to reveal genetic characteristics of the GA breed. As a novel chicken breed, GA breed displays population structure clearly separated from other chicken breeds based on overall genomic variants and TE insertion information. Furthermore, we identified candidate genes which could provide insight into the association between genetic traits and phenotypic distinctions such as large body size, flight ability, egg production, and blue egg shell formation using various measures. This information and the sequencing data used in this study could be invaluable for understanding genomic features of a novel breed which has not been previously studied, as well as provide a basis for selective breeding of traits crucial to the poultry production industry.

## Methods

### Ethics statement

The experiment and all its procedures were approved by the regional Ethical Committee (Gyeong Buk Animal Bioethics committee permit number: 2012-0049), and the methods were carried out in accordance with the approved guidelines.

### DNA sequencing and sample collection

We obtained blood samples from male GA, LH, and KNC chickens. Samples were collected from the Livestock Research Institute, Yeongju, Korea. To prevent clotting, blood drawn from the carotid artery was treated with heparin. To generate inserts of ~300 bp fragments, 3 μg of genomic DNA was randomly sheared using Covaris System. The TruSeq Dna Sample Prep. Kit (Illumina, San Diego, CA) was used for library construction following manufacturer guidelines. Whole genome sequencing was performed using the Illumina HiSeq 2000 platform. Additionally, we downloaded genomic data of 14 chicken samples from the EMBL-EBI database, which included 2 Silkies (one from China and one from Taiwan), 1 Taiwanese native chicken (TNC), 1 Leghorn, 1 Tibetan chicken (TB), 1 Shouguang (SG), 1 Wenchang (WC), 1 Beijing You (BY), 1 White Plymouth Rock (WPR), 1 Dong Xiang (DX), 1 Cornish (CN), 1 Luxi Game (LG), 1 Rhode Island Red (RIR), and 1 Red Jungle Fowl (RJF). Also, data from 5 Korean Native Chickens, used in a previous study, were included to increase the quality of variants calling and following analyses (Table S5). A quality check on raw sequence data was performed using fastQC^37^ software, and potential adapter sequences were removed prior to sequence alignment using Trimmomatic-0.32^38^.

### Short reads alignment and variants calling

Paired-end sequence reads were mapped to the chicken reference genome (*Galgal 4.75*) from the Ensembl database using Bowtie2^39^ with default settings. For downstream processing and variant calling, we used several open-source software packages: Picard tools (http://picard.sourceforge.net), SAMtools^40^, and Genome Analysis Toolkit (GATK)^41^. “CreateSequenceDictionary” and “MarkDuplicates” Picard command-line tools were used to read reference FASTA sequence for writing bam file with only sequence dictionary, and to filter potential PCR duplicates, respectively. Using SAMtools, we created index files for the reference and bam files. We then performed local realignment of sequence reads to correct misalignment due to the presence of small insertion and deletion using GATK “RealignerTargetCreator” and “IndelRealigner” arguments. Base quality score recalibration was performed to get accurate quality scores and to correct the variation in quality with machine cycle and sequence context. For variant calling, GATK “UnifiedGenotyper” and “SelectVariants” arguments were used with the following filtering criteria: all variants with 1) a Phred-scaled quality score of less than 30; 2) read depth less than 5; 3) MQ0 (total count across all samples of mapping quality zero reads) >4; or a 4) Phred-scaled P-value using Fisher’s exact test more than 200 were filtered out to reduce false positive calls due to the strand bias. Annotation of the calling variants were performed using Snpeff^42^ with default settings.

### Short reads assembly and functional annotation

We used the “Error correction” module of Allpaths-LG^43^ with default settings to eliminate possible sequencing errors. Error-corrected paired-end reads were merged into FASTA format using “Fq2fa” module from IDBA v1.1.1 software which stands for iterative De Bruijn graph De novo assembler for short reads sequencing data with highly uneven sequencing depth. We assembled error corrected paired-end reads using IDBA_UD from IDBA package^44^ with the following parameters: 1) Perform pre-correction before assembly (“–pre_correction”), and 2) minimum k value should be more than 30 (–mink 30). Using Gapcloser^45^, we filled predicted gaps in the assembled sequences using default settings. RepeatMasker^46^ was used to screens DNA sequences for interspersed repeats and low complexity DNA sequences before gene prediction for the candidate contigs. We matched the unmapped assembled contigs to the whole genome assembled contigs using BLAST, and potential coding regions in the contigs sequence were predicted using AUGUSTUS^47^. Coding regions were aligned to chicken reference peptide sequences (Galgal 4.75) from the Ensembl database using BLASTP for functional annotation. A detailed schematization of this process is shown in Figure S5.

### Population stratification and selective sweep analysis

We used Genome-Wide Complex Trait Analysis (GCTA)^48^ to calculate eigenvectors, which are equivalent to those estimated by the EIGENSTRAT software tool for principal component analysis (PCA). Autosomal genotype data was converted to PLINK format, the input format required for GCTA, using VCFtools^49^.

To investigate linkage disequilibrium patterns, the coefficient of determination (r^2^) between any two SNVs was calculated using Haploview^50^. The average of pairwise r^2^ was calculated according to the distance between two SNVs in a 500 kb window and averaged for the whole genome.

Pairwise relatedness and inbreeding coefficient were measured using the KING program^51^ command with parameter ‘–kinship –ibs’. VCFtools 4.0^49^ was used to compute nucleotide variation (*θ*_*π*_) and genetic differentiation (F_ST_) by applying a sliding window approach (bin size 100 kb, step size 20 kb).

### Transposable element (TE) probes

As the majority of TEs in the chicken genome are of the retrotransposon type^52^, we focused on four types of retrotransposons in this study: LTR, LINE, SINE, and DNA transposons. Thus, we obtained all possible TE probe sequences from Repeatmasker Genomic datasets (http://www.repeatmasker.org/) and used these as the alignment subjects for TE identification. The coordinates of TEs from the Gallus gallus reference genome (from Repeat library 4, 20140131) were also used TE identification.

### Transposable element variants (TEV) identification

We identified TE variants (TEV) across 28 chicken genomes using Retroseq software^22^. Retroseq employs discordantly or solely mapping reads to seek candidate TE insertion sites, called breakpoints. In the alignment step, it was necessary to determine the appropriate insert size of paired reads to obtain pure discordantly mapping reads induced by TE insertion not by the mapping distance. We set the minimum insert size (1000 bp) to guarantee this requisite.

Due to the difference in depth coverage across the paired-end reads data from various data production processes, it was necessary to adjust the parameters for each group for each different data production process. Additionally, for the confidence of breakpoint, we filtered the raw calls of TEVs and only recovered the calls tagged as “FL = 8” which met all breakpoint criteria. Final calls of TEVs were classified into four groups (LTR, LINE, SINE, and DNA transposons) based on the nomenclature and classification used in Repeatmasker. For population analyses, TEV calls within 100 bps were clustered and regarded as a single TEV call; using these cluster positions, we identified breed-specific breakpoint and related genes.

### Clustering analysis based on TE presence polymorphism

Using cluster position, presence of a TE insertion was coded as “1” or “2” according to genotypes, and absence of TE insertion was coded as “0” for each individual. To improve the reliability of each TE insertion loci, loci present in less than 3 individual genomes were excluded. This data matrix was used as an input for calculation of p-distance matrix calculated by the hamming distance metric. We implemented the neighbor-joining method and bootstrapped the data using the phangorn^53^ package within R to cluster samples.

## Additional Information

**Accessions codes**: The samples that were sequenced were archived at the Sequence Read Archive (SRA) (http://www.ncbi.nlm.nih.gov/sra/) under the accession SRP051746.

**How to cite this article**: Jeong, H. *et al*. Whole genome sequencing of Gyeongbuk Araucana, a newly developed blue-egg laying chicken breed, reveals its origin and genetic characteristics. *Sci. Rep*. **6**, 26484; doi: 10.1038/srep26484 (2016).

## Supplementary Material

Supplementary Information

## Figures and Tables

**Figure 1:**
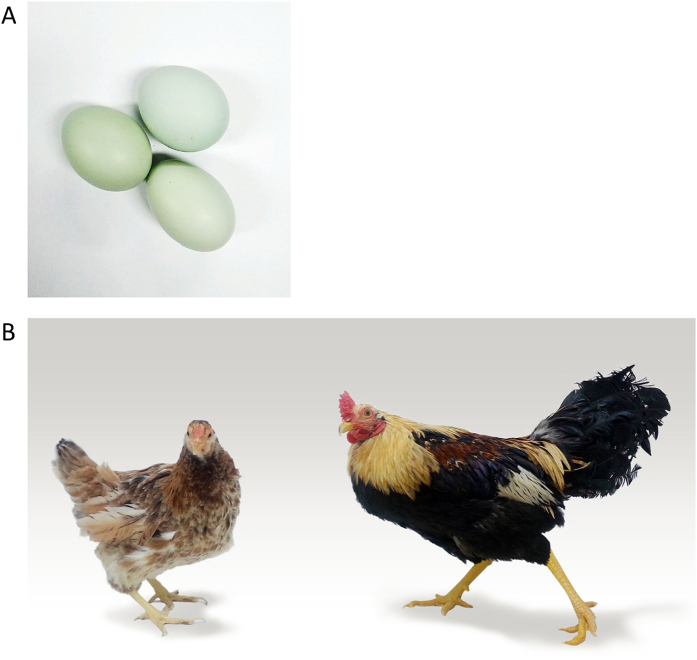
Gyeongbuk Araucana chicken. (**A**) blue egg shell of Gyeongbuk Araucana. (**B**) general appearance of Gyeongbuk Araucana chicken breed.

**Table 1:** Results of genetic variants annotation using Snpeff.

	Gyeongbuk Araucana		Leghorn		Korean native Chicken	
	SNP	INDEL	SNP	INDEL	SNP	INDEL
Region type						
Downstream	641519	112867	655710	114991	1007516	152666
Exon	96037	2051	98252	2017	150948	2340
Intergenic	3827999	712665	3923139	726943	6084648	973939
Intron	3418642	633210	3501287	646854	5369842	867038
Splice site acceptor	145	136	147	129	226	152
Splice site donor	184	152	184	143	289	166
Splice site region	12613	2580	12810	2611	19885	3377
Upstream	656713	106253	668732	107865	1014086	141518
UTR 3′	75671	17685	77469	18094	120027	23795
UTR 5′	15091	1965	14885	1883	20808	2056
Functional class						
Missense	25167	—	25851	—	40408	—
Nonsense	175	—	173	—	293	—
Silent	69784	—	71254	—	108748	—
Total	7124664	1312246	7304278	1339702	11275071	1794984

A summary of results for other chicken breeds is shown in Table S2.

**Table 2:** Results of transposable elements variants annotation using Snpeff.

Region Type	Gyeongbuk Araucana	Leghorn	Korean native Chicken
Downstream	2067	4174	14619
Exon	11	28	48
Intergenic	14059	30101	128283
Intron	7890	18947	91577
None	17	21	36
Splice site acceptor	4	13	33
Splice site donor	7	14	29
Splice site region	44	81	288
Upstream	2688	5169	14997
UTR 3′	156	292	1298
UTR 5′	108	207	236
Total	22033	48797	216315

A summary of results for other chicken breeds is shown in Table S4.
