# Supplementary material for: Whole genome sequencing of Gyeongbuk Araucana, a newly developed blue-egg laying chicken breed, reveals its origin and genetic characteristics
Source: Sci Rep. 2016 May 24;6:26484. doi: 10.1038/srep26484 (PMC4877703; doi:10.1038/srep26484)
Supplement: Supplementary Information [file srep26484-s1.doc]

**Supplementary Information for**

**Whole genome sequencing of Gyeongbuk Araucana, a newly developed blue-egg laying chicken breed, reveals its origin and genetic characteristics**

Hyeonsoo Jeong^1, †^, Kwondo Kim^2, 6, †^, Kelsey Caetano-Anollés^3^, Heebal Kim^2, 4^, Byung-ki Kim^5^, Jun-Koo Yi^5^, Jae-Jung Ha^5^, Seoae Cho^6^,*, Dong Yep Oh^5^,*

^1^Department of Animal Sciences, University of Illinois, Urbana, IL 61801, USA

^2^Interdisciplinary Program in Bioinformatics, Seoul National University, Kwan-ak St. 599, Kwan-ak Gu, Seoul, South Korea 151-741,Republic of Korea.

^3^Department of Agricultural Biotechnology, Animal Biotechnology Major, and Research Institute for Agriculture and Life Sciences, Seoul National University, Seoul 151-921, Korea

^4^Department of Agricultural Biotechnology, Seoul National University, Kwan-ak St. 599, Kwan-ak Gu, Seoul 151-742, Republic of Korea

^5^Gyeongsangbuk-do Livestock Research Institute, 186, Daeryongsan-ro, Anjung-myon, Yeoungju, Gyeongsangbuk-do, Republic of Korea

^6^C&K genomics, Main Bldg. #514, SNU Research Park, Seoul 151-919, Republic of Korea

^†^ These authors equally contributed and should be regarded as co-first authors.

*Corresponding author:

Seoae Cho, Tel) +82 (2)880 8820, Fax) +82(2)876 8827, Email: seoae@cnkgenomics.com

Dong Yep Oh, Tel) +82(54)638-6012, Email: ody1234@korea.kr

**Table of contents**

**Supplementary Fig. S1-S6**

Supplementary Fig. S1 | The distributions of novel SNVs and known SNPs in each chromosome.

Supplementary Fig. S2 | The ratios of the number of SNVs (left) and InDel in each chromosome to the total number of variants.

Supplementary Fig. S3 | The pairwise relationship between each sample using kinship coefficient and IBS (identical by state).

Supplementary Fig. S4 | Clustering trees based on (A) SNVs, (B) DNA transposons, (C) LINE, and (D) SINE transposons.

Supplementary Fig. S5 | A detailed schematization of the short reads assembly process.

**Supplementary Tables S1-S5**

Supplementary Table S1 | Summary statistics of sequence read mapping results using Bowtie2.

Supplementary Table S2 | Summary of results of genetic variants annotation using Snpeff.

Supplementary Table S3 | Summary statistics of assembled contigs for the GA breed using IDBA_UD.

Supplementary Table S4 | Summary of results of transposable element variants annotation using Snpeff.

Supplementary Table S5 | Information on the samples acquired from public databases.


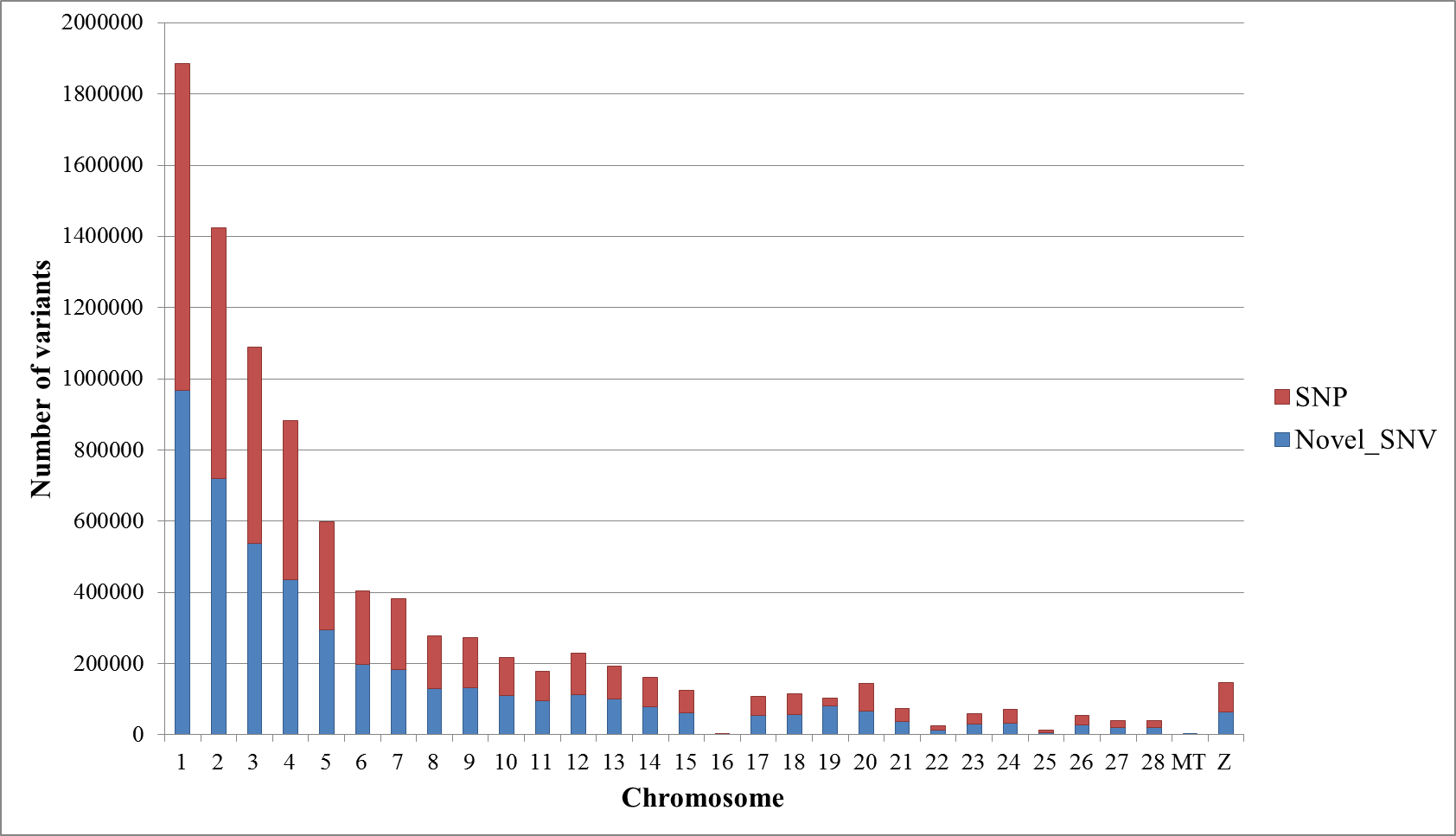


**Supplementary Fig. S1 | The distribution of novel SNVs and known SNPs in each chromosome.**


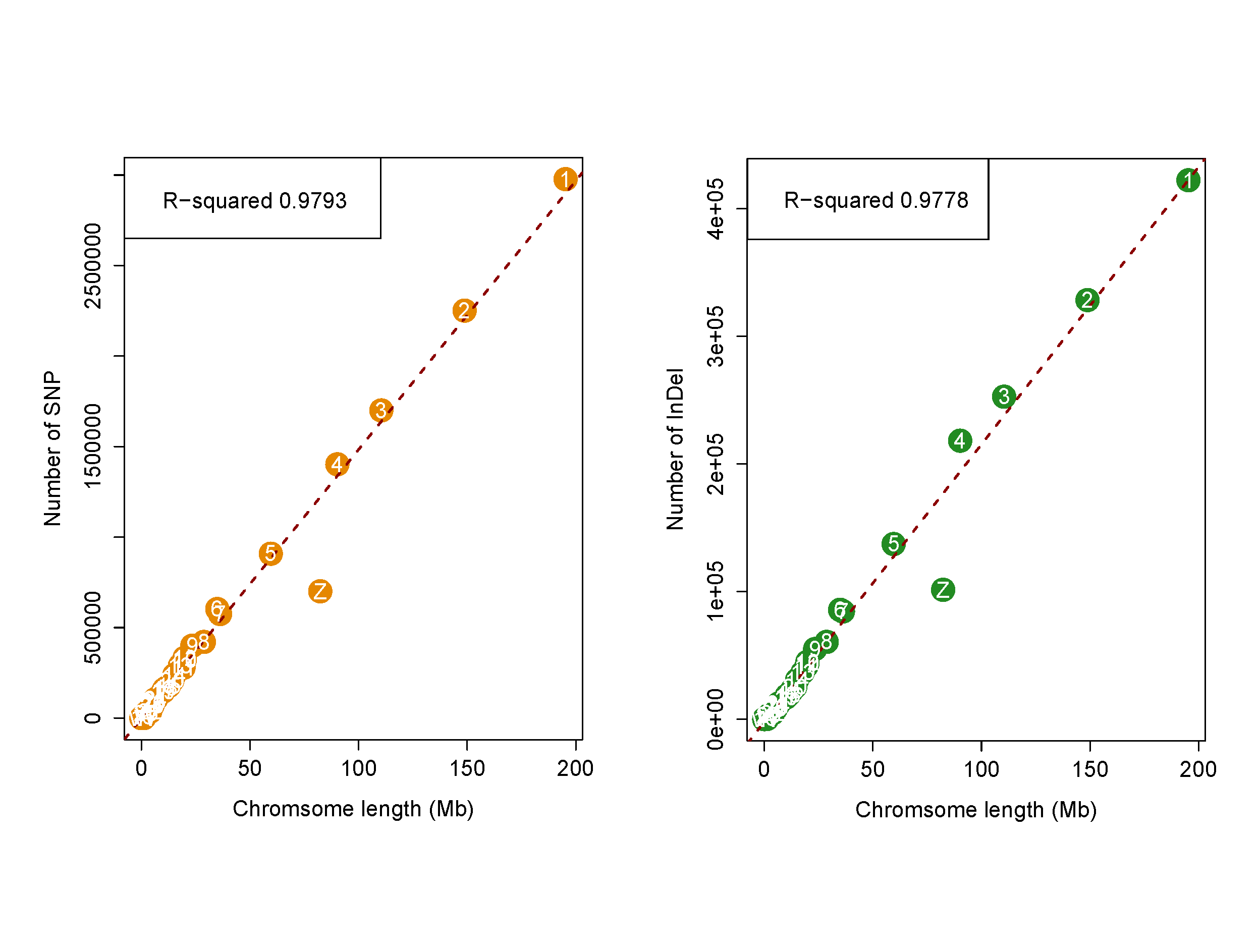


**Supplementary Fig. S2 | The ratios of the number of SNVs (left) and InDel in each chromosome to the total number of variants.** Z chromosomes have been shown to contain few genomic variants compared to other chromosomes, which supports the idea that the Z chromosome in avian species has been highly conserved during evolution [^1^](#_ENREF_1).


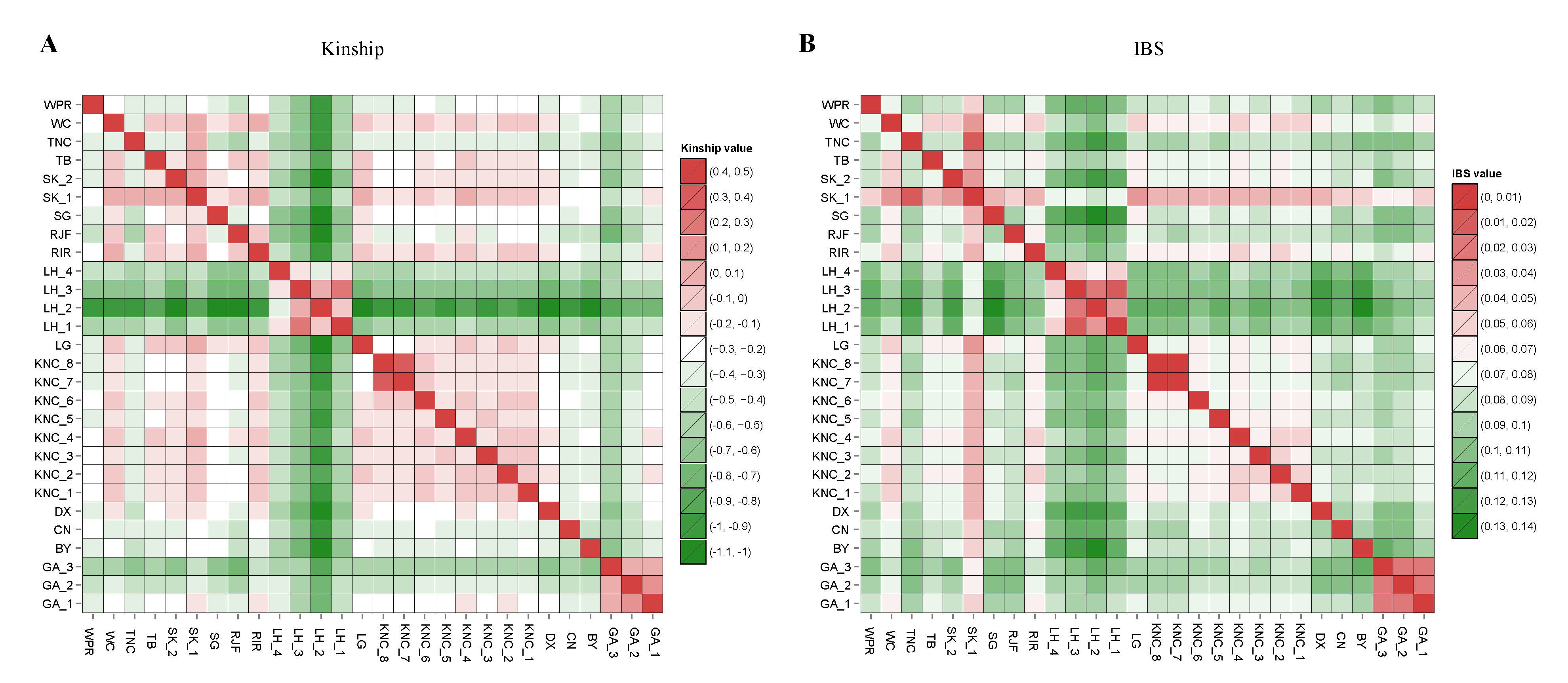


**Supplementary Fig. S3 | The pairwise relationship between each sample using kinship coefficient and IBS (identical by state).**





**Supplementary Fig. S4 | Clustering trees based on (A) SNVs, (B) DNA transposons, (C) LINE, and (D) SINE transposons.**


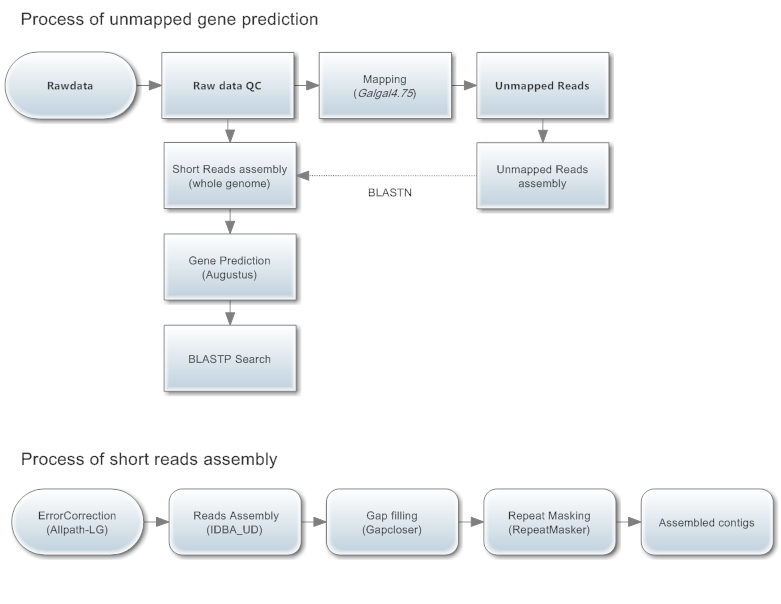


**Supplementary Fig. S5 | A detailed schematization of the short read assembly process.**

**Supplementary Table S1. Summary statistics of sequence read mapping results using Bowtie2 (GA, KNC, and LH breeds).**

| Samples | Total number of reads | Mapped reads | Mapped reads (both in pair) | Unmapped reads | Duplication rate |
| --- | --- | --- | --- | --- | --- |
| GA_1 | 127,501,128 | 124,789,693 / 97.87% | 123,911,348 / 97.18% | 2,711,435 / 2.13% | 6.81% |
| GA_2 | 128,522,316 | 125,674,468 / 97.78% | 124,867,180 / 97.16% | 2,847,848 / 2.22% | 6.88% |
| GA_3 | 102,586,645 | 100,316,324 / 97.79% | 99,599,442 / 97.09% | 2,270,321 / 2.21% | 5.63% |
| KNC_6 | 141,210,800 | 138,593,259 / 98.15% | 137,666,110 / 97.49% | 2,617,541 / 1.85% | 7.43% |
| KNC_7 | 109,216,135 | 106,970,436 / 97.94% | 105,979,082 / 97.04% | 2,245,699 / 2.06% | 6.02% |
| KNC_8 | 104,075,162 | 102,289,174 / 98.28% | 101,624,186 / 97.64% | 1,785,988 / 1.72% | 5.78% |
| LH_1 | 149,021,705 | 146,225,242 / 98.12% | 145,253,640 / 97.47% | 2,796,463 / 1.88% | 8.16% |
| LH_2 | 127,801,277 | 125,561,647 / 98.25% | 124,748,492 / 97.61% | 2,239,630 / 1.75% | 6.87% |
| LH_3 | 105,209,212 | 103,258,060 / 98.15% | 102,522,238 / 97.45% | 1,951,152 / 1.85% | 5.78% |
| Total | 1,095,144,380 | 1,073,678,303 / 98.03% | 1,066,171,718 / 97.35% | 21,466,077 / 1.96% | 6.60% |

* Estimated chicken genome size: 1 Gb
* Fastq Quality Encoding: Sanger / Illumina 1.9 encoding

**Supplementary Table S1 (cont.). Summary statistics of sequence read mapping results using Bowtie2 (downloaded chicken genome data**).

| Samples | Total number of reads | Mapped reads | Mapped reads (both in pair) | Unmapped reads | Duplication rate |
| --- | --- | --- | --- | --- | --- |
| BY | 114,486,819 | 112,200,881 / 98% | 111,712,700 / 97.58% | 2,285,938 / 2% | 6.59% |
| CN | 94,855,251 | 92,769,468 / 97.8% | 92,335,236 / 97.34% | 2,085,783 / 2.2% | 5.59% |
| DX | 142,074,176 | 139,417,840 / 98.13% | 138,810,676 / 97.7% | 2,656,336 / 1.87% | 7.96% |
| KNC_1 | 353,890,779 | 345,386,688 / 97.6% | 342,455,340 / 96.77% | 8,504,091 / 2.4% | 17.62% |
| KNC_2 | 334,722,059 | 326,245,912 / 97.47% | 323,096,796 / 96.53% | 8,476,147 / 2.53% | 16.72% |
| KNC_3 | 353,045,229 | 344,291,172 / 97.52% | 341,327,614 / 96.68% | 8,754,057 / 2.48% | 17.42% |
| KNC_4 | 334,300,445 | 325,671,816 / 97.42% | 322,604,520 / 96.5% | 8,628,629 / 2.58% | 17.32% |
| KNC_5 | 362,370,741 | 351,766,520 / 97.07% | 347,806,098 / 95.98% | 10,604,221 / 2.93% | 17.44% |
| LH_4 | 128,488,999 | 126,032,220 / 98.09% | 125,477,202 / 97.66% | 2,456,779 / 1.91% | 6.81% |
| LG | 118,032,962 | 115,425,741 / 97.79% | 114,903,456 / 97.35% | 2,607,221 / 2.21% | 6.78% |
| RIR | 142,072,198 | 138,814,947 / 97.71% | 137,933,728 / 97.09% | 3,257,251 / 2.29% | 19.09% |
| RJF | 117,335,138 | 114,152,693 / 97.29% | 113,524,112 / 96.75% | 3,182,445 / 2.71% | 8.66% |
| SG | 96,033,628 | 93,764,207 / 97.64% | 93,320,666 / 97.17% | 2,269,421 / 2.36% | 5.78% |
| SK_1 | 227,666,842 | 218,310,740 / 95.89% | 216,501,912 / 95.1% | 9,356,102 / 4.11% | 12.74% |
| SK_2 | 105,321,972 | 103,042,008 / 97.84% | 102,578,718 / 97.4% | 2,279,964 / 2.16% | 6.08% |
| TB | 117,249,612 | 115,306,132 / 98.34% | 114,825,404 / 97.93% | 1,943,480 / 1.66% | 6.74% |
| TNC | 262,800,932 | 248,007,720 / 94.37% | 244,909,716 / 93.19% | 14,793,212 / 5.63% | 13.98% |
| WC | 129,072,209 | 127,027,406 / 98.42% | 126,497,914 / 98.01% | 2,044,803 / 1.58% | 7.43% |
| WPR | 140,186,905 | 137,649,206 / 98.19% | 137,074,260 / 97.78% | 2,537,699 / 1.81% | 7.36% |
| Total | 3,674,006,896 | 3,575,283,317 / 97.50% | 3,547,696,068 / 96.87% | 73,326,146 / 2.05% | 10.95% |

* Estimated chicken genome size: 1 Gb
* Fastq Quality Encoding: Sanger / Illumina 1.9 encoding

**Supplementary Table S2. Summary of results of genetic variants annotation using Snpeff.**

|  | GA | | BY | | CN | | DX | | KNC | |
| --- | --- | --- | --- | --- | --- | --- | --- | --- | --- | --- |
|  | SNP | INDEL | SNP | INDEL | SNP | INDEL | SNP | INDEL | SNP | INDEL |
| **Region type** |  |  |  |  |  |  |  |  |  |  |
| Downstream | 641,519 | 112,867 | 494,360 | 88,974 | 490,471 | 88,118 | 512,979 | 91,850 | 1,007,516 | 152,666 |
| Exon | 96,037 | 2,051 | 73,930 | 1,726 | 72,627 | 1,705 | 76,710 | 1,743 | 150,948 | 2,340 |
| Intergenic | 3,827,999 | 712,665 | 2,953,388 | 559,131 | 2,899,493 | 549,967 | 3,064,855 | 576,639 | 6,084,648 | 973,939 |
| Intron | 3,418,642 | 633,210 | 2,625,382 | 495,949 | 2,579,937 | 487,505 | 2,724,848 | 508,474 | 5,369,842 | 867,038 |
| Splice site acceptor | 145 | 136 | 113 | 127 | 129 | 129 | 132 | 127 | 226 | 152 |
| Splice site donor | 184 | 152 | 139 | 140 | 136 | 143 | 140 | 145 | 289 | 166 |
| Splice site region | 12,613 | 2,580 | 9,769 | 2,027 | 9,699 | 2,037 | 10,114 | 2,075 | 19,885 | 3,377 |
| Upstream | 656,713 | 106,253 | 501,427 | 83,288 | 498,514 | 82,739 | 524,567 | 86,703 | 1,014,086 | 141,518 |
| UTR 3’ | 75,671 | 17,685 | 57,099 | 13,628 | 56,768 | 13,539 | 59,696 | 13,976 | 120,027 | 23,795 |
| UTR 5’ | 15,091 | 1,965 | 10,992 | 1,433 | 10,666 | 1,364 | 11,606 | 1,524 | 20,808 | 2,056 |
| **Functional class** |  |  |  |  |  |  |  |  |  |  |
| Missense | 25,167 | - | 19,094 | - | 18,712 | - | 19,886 | - | 40,408 | - |
| Nonsense | 175 | - | 127 | - | 116 | - | 135 | - | 293 | - |
| Silent | 69,784 | - | 54,017 | - | 53,123 | - | 55,956 | - | 108,748 | - |
| Total | 7,124,664 | 1,312,246 | 5,487,281 | 1,029,026 | 5,391,948 | 1,012,063 | 5,694,311 | 1,058,550 | 11,275,071 | 1,794,984 |

**Supplementary Table S2 (cont.). Summary of results of genetic variants annotatio**n using Snpeff.

|  | LH | | LG | | RIR | | RJF | | SG | |
| --- | --- | --- | --- | --- | --- | --- | --- | --- | --- | --- |
|  | SNP | INDEL | SNP | INDEL | SNP | INDEL | SNP | INDEL | SNP | INDEL |
| **Region type** |  |  |  |  |  |  |  |  |  |  |
| Downstream | 655,710 | 114,991 | 534,288 | 93,516 | 529,409 | 91,486 | 565,351 | 94,969 | 496,600 | 88,895 |
| Exon | 98,252 | 2,017 | 79,189 | 1,773 | 78,593 | 1,631 | 83,600 | 1,711 | 73,994 | 1,654 |
| Intergenic | 3,923,139 | 726,943 | 3,178,189 | 580,946 | 3,225,966 | 579,391 | 3,424,118 | 594,818 | 2,980,259 | 561,472 |
| Intron | 3,501,287 | 646,854 | 2,814,605 | 512,203 | 2,867,463 | 515,872 | 3,057,124 | 530,944 | 2,675,987 | 497,573 |
| Splice site acceptor | 147 | 129 | 133 | 128 | 126 | 129 | 147 | 128 | 110 | 133 |
| Splice site donor | 184 | 143 | 152 | 149 | 151 | 138 | 139 | 144 | 138 | 145 |
| Splice site region | 12,810 | 2,611 | 10,583 | 2,126 | 10,596 | 2,105 | 11,220 | 2,254 | 9,758 | 2,075 |
| Upstream | 668,732 | 107,865 | 544,252 | 87,640 | 537,833 | 84,965 | 574,096 | 88,220 | 504,442 | 83,036 |
| UTR 3’ | 77,469 | 18,094 | 62,148 | 14,039 | 62,143 | 13,876 | 66,655 | 14,679 | 58,103 | 13,653 |
| UTR 5’ | 14,885 | 1,883 | 11,815 | 1,537 | 11,325 | 1,450 | 11,816 | 1,478 | 10,656 | 1,325 |
| **Functional class** |  |  |  |  |  |  |  |  |  |  |
| Missense | 25,851 | - | 20,553 | - | 20,291 | - | 21,336 | - | 19,105 | - |
| Nonsense | 173 | - | 141 | - | 138 | - | 137 | - | 121 | - |
| Silent | 71,254 | - | 57,727 | - | 57,426 | - | 61,338 | - | 54,075 | - |
| Total | 7,304,278 | 1,339,702 | 5,893,407 | 1,065,876 | 5,990,242 | 1,067,188 | 6,372,565 | 1,097,703 | 5,562,241 | 1,033,195 |

**Supplementary Table S2 (cont.). Summary of results of genetic variants** annotation using Snpeff.

|  | SK | | TNC | | TB | | WC | | WPR | |
| --- | --- | --- | --- | --- | --- | --- | --- | --- | --- | --- |
|  | SNP | INDEL | SNP | INDEL | SNP | INDEL | SNP | INDEL | SNP | INDEL |
| **Region type** |  |  |  |  |  |  |  |  |  |  |
| Downstream | 758,307 | 122,384 | 506,274 | 93,556 | 545,447 | 93,977 | 551,125 | 94,817 | 496,379 | 91,224 |
| Exon | 114,510 | 2,164 | 75,589 | 1,897 | 80,873 | 1,808 | 82,421 | 1,835 | 74,110 | 1,746 |
| Intergenic | 4,523,089 | 765,876 | 2,930,182 | 567,251 | 3,224,750 | 585,515 | 3,273,545 | 591,941 | 2,957,513 | 572,504 |
| Intron | 3,996,184 | 680,660 | 2,596,493 | 496,139 | 2,867,267 | 519,474 | 2,917,113 | 524,503 | 2,615,664 | 500,304 |
| Splice site acceptor | 166 | 145 | 117 | 136 | 133 | 137 | 134 | 124 | 125 | 131 |
| Splice site donor | 226 | 157 | 160 | 154 | 169 | 149 | 148 | 152 | 143 | 135 |
| Splice site region | 15,041 | 2,708 | 9,853 | 2,154 | 10,781 | 2,189 | 10,968 | 2,183 | 9,878 | 2,101 |
| Upstream | 775,193 | 115,384 | 519,441 | 88,589 | 555,448 | 88,400 | 562,980 | 89,312 | 509,540 | 86,456 |
| UTR 3’ | 89,115 | 18,528 | 58,435 | 14,107 | 62,993 | 14,074 | 64,258 | 14,143 | 58,009 | 14,030 |
| UTR 5’ | 17,184 | 1,965 | 12,154 | 1,770 | 11,944 | 1,557 | 12,404 | 1,585 | 11,563 | 1,608 |
| **Functional class** |  |  |  |  |  |  |  |  |  |  |
| Missense | 30,093 | - | 19,836 | - | 20,967 | - | 21,333 | - | 19,239 | - |
| Nonsense | 209 | - | 132 | - | 143 | - | 156 | - | 116 | - |
| Silent | 83,117 | - | 54,927 | - | 59,014 | - | 60,171 | - | 54,062 | - |
| Total | 8,385,758 | 1,410,519 | 5,439,270 | 1,038,220 | 5,996,295 | 1,077,423 | 6,091,098 | 1,088,681 | 5,483,994 | 1,046,795 |

**Supplementary Table S3. Summary statistics of assembled contigs using IDBA_UD.**

| Sample name | GA_1 assembled contigs | GA_2 assembled contgs | GA_3 assembled contigs |
| --- | --- | --- | --- |
| **Number of contigs** | 81535 | 79143 | 123326 |
| **Sequence lengths** |  |  |  |
| Minimum length | 2000 | 2000 | 2000 |
| Maximum length | 188506 | 181240 | 67713 |
| Average length | 10895.81 | 11360.3 | 5964.42 |
| N50 length | 16252 | 17059 | 7353 |
| **Residue contents** |  |  |  |
| Total residue counts | 888390003 | 899088552 | 735567501 |
| GC contents | 360839748 / 40.61% | 365231271 / 40.62% | 294894634 / 40.09% |
| N contents | 1295693 / 0.15% | 993308 / 0.11% | 4901193 / 0.67% |
| Closed N by Gapcloser | 355825 | 263962 | 689213 |

**Supplementary Table S3 (cont.). Summary statistics of assembled contigs using IDBA_UD.**

| Sample name | LH_1 assembled contigs | LH_2 assembled contgs | LH_3 assembled contigs |
| --- | --- | --- | --- |
| **Number of contigs** | 70452 | 96358 | 128044 |
| **Sequence lengths** |  |  |  |
| Minimum length | 2000 | 2000 | 2000 |
| Maximum length | 235548 | 127425 | 56592 |
| Average length | 12842.4 | 8730.7 | 4911.94 |
| N50 length | 20563 | 12450 | 5657 |
| **Residue contents** |  |  |  |
| Total residue counts | 904769060 | 841270043 | 628944712 |
| GC contents | 367059265 / 40.57% | 338001953 / 40.18% | 248036683 / 39.44% |
| N contents | 671615 / 0.07% | 1243035 / 0.15% | 2748954 / 0.44% |
| Closed N by Gapcloser | 218001 | 253919 | 395172 |

**Supplementary Table S3 (cont.). Summary statistics of assembled contigs using IDBA_UD.**

| Sample name | KNC_1 assembled contigs | KNC_2 assembled contgs | KNC_3 assembled contigs |
| --- | --- | --- | --- |
| **Number of contigs** | 81307 | 117135 | 131478 |
| **Sequence lengths** |  |  |  |
| Minimum length | 2000 | 2000 | 2000 |
| Maximum length | 179711 | 87459 | 48960 |
| Average length | 10811.8 | 5553.7 | 4586.5 |
| N50 length | 16440 | 6698 | 5138 |
| **Residue contents** |  |  |  |
| Total residue counts | 879071004 | 650528051 | 603028811 |
| GC contents | 354514706 / 40.33% | 254748024 / 39.16% | 239898363 / 39.78% |
| N contents | 1333258 / 0.15% | 4802272 / 0.74% | 2327240 / 0.39% |
| Closed N by Gapcloser | 360704 | 949123 | 387760 |

**Supplementary Table S4. Summary of results** of transposable element variants annotation using Snpeff.

| Region Type | GA | BY | CN | DX | KNC | LH | LG | RIR |
| --- | --- | --- | --- | --- | --- | --- | --- | --- |
| Downstream | 2,067 | 727 | 704 | 968 | 14,619 | 4,174 | 866 | 659 |
| Exon | 11 | 3 | 2 | 2 | 48 | 28 | 2 | 3 |
| Intergenic | 14,059 | 5,715 | 4,803 | 7,724 | 128,283 | 30,101 | 6,364 | 5,024 |
| Intron | 7,890 | 3,693 | 3,067 | 5,034 | 91,577 | 18,947 | 3,951 | 2,842 |
| None | 17 | 1 | 1 | 5 | 36 | 21 | 1 | 3 |
| Splice site acceptor | 4 | 4 | 3 | 4 | 33 | 13 | 7 | 1 |
| Splice site donor | 7 | 1 | 0 | 3 | 29 | 14 | 2 | 2 |
| Splice site region | 44 | 11 | 9 | 24 | 288 | 81 | 20 | 10 |
| Upstream | 2,688 | 910 | 736 | 1,106 | 14,997 | 5,169 | 970 | 706 |
| UTR 3’ | 156 | 38 | 41 | 73 | 1,298 | 292 | 66 | 53 |
| UTR 5’ | 108 | 17 | 6 | 28 | 236 | 207 | 19 | 13 |
| Total | 22,033 | 9,371 | 7,825 | 12,671 | 216,315 | 48,797 | 10,251 | 7,887 |

**Supplementary Table S4 (cont.). Summary of results** of transposable element variants annotation using Snpeff.

| Region Type | RJF | SG | SK | TB | TNC | WC | WPR |
| --- | --- | --- | --- | --- | --- | --- | --- |
| Downstream | 908 | 648 | 2,296 | 829 | 1,984 | 849 | 1,126 |
| Exon | 4 | 5 | 16 | 3 | 15 | 5 | 7 |
| Intergenic | 7,237 | 5,282 | 10,800 | 6,087 | 7,263 | 6,672 | 8,838 |
| Intron | 4,548 | 3,300 | 6,154 | 3,753 | 3,613 | 4,239 | 5,557 |
| None | 0 | 3 | 11 | 2 | 16 | 3 | 3 |
| Splice site acceptor | 0 | 0 | 6 | 0 | 3 | 6 | 5 |
| Splice site donor | 2 | 0 | 3 | 2 | 5 | 3 | 2 |
| Splice site region | 14 | 10 | 36 | 13 | 16 | 18 | 21 |
| Upstream | 976 | 698 | 2,838 | 898 | 2,701 | 1,038 | 1,319 |
| UTR 3’ | 50 | 49 | 149 | 49 | 67 | 58 | 92 |
| UTR 5’ | 29 | 3 | 107 | 14 | 82 | 27 | 39 |
| Total | 11,690 | 8,518 | 17,176 | 9,791 | 10,912 | 10,880 | 14,395 |

**Supplementary Table S5. Information on the samples acquired from public databases.**

| Chicken breeds (number of samples) | Accession number | Reference |
| --- | --- | --- |
| White Leghon (1) | SRA123124 | [^2^](#_ENREF_2) |
| Beijing You (1) | SRA123124 | [^2^](#_ENREF_2) |
| Cornish (1) | SRA123124 | [^2^](#_ENREF_2) |
| Dongxiang (1) | SRA123124 | [^2^](#_ENREF_2) |
| Luxi Game (1) | SRA123124 | [^2^](#_ENREF_2) |
| Rhode Island Red (1) | SRA123124 | [^2^](#_ENREF_2) |
| Red Jungle Fowl (1) | SRA123124 | [^2^](#_ENREF_2) |
| Shouguang (1) | SRA123124 | [^2^](#_ENREF_2) |
| Silky (China) (1) | SRA123124 | [^2^](#_ENREF_2) |
| Silky (Taiwan) (1) | SRA080358 | [^3^](#_ENREF_3) |
| Tibetan (1) | SRA123124 | [^2^](#_ENREF_2) |
| Wenchang (1) | SRA123124 | [^2^](#_ENREF_2) |
| White Plymouth Rock (1) | SRA123124 | [^2^](#_ENREF_2) |
| Korean Native Chicken (5) | SRP040256 | [^4^](#_ENREF_4) |

**References**

1 Nanda, I. *et al.* 300 million years of conserved synteny between chicken Z and human chromosome 9. *Nat Genet* **21**, 258-259 (1999).

2 Yi, G. *et al.* Genome-wide patterns of copy number variation in the diversified chicken genomes using next-generation sequencing. *BMC genomics* **15**, 962 (2014).

3 Fan, W.-L. *et al.* Genome-wide patterns of genetic variation in two domestic chickens. *Genome. Biol. Evol.* **5**, 1376-1392 (2013).

4 Kwak, W. *et al.* Uncovering Genomic Features and Maternal Origin of Korean Native Chicken by Whole Genome Sequencing. *PLoS. ONE* **9**, e114763 (2014).
